# Forecasting and trading cryptocurrencies with machine learning under changing market conditions

**DOI:** 10.1186/s40854-020-00217-x

**Published:** 2021-01-06

**Authors:** Helder Sebastião, Pedro Godinho

**Affiliations:** Univ Coimbra, CeBER, Faculty of Economics, Av. Dr. Dias da Silva, 165, 3004-512 Coimbra, Portugal

**Keywords:** Bitcoin, Ethereum, Litecoin, Machine learning, Forecasting, Trading, G11, G15, G17

## Abstract

This study examines the predictability of three major cryptocurrencies—bitcoin, ethereum, and litecoin—and the profitability of trading strategies devised upon machine learning techniques (e.g., linear models, random forests, and support vector machines). The models are validated in a period characterized by unprecedented turmoil and tested in a period of bear markets, allowing the assessment of whether the predictions are good even when the market direction changes between the validation and test periods. The classification and regression methods use attributes from trading and network activity for the period from August 15, 2015 to March 03, 2019, with the test sample beginning on April 13, 2018. For the test period, five out of 18 individual models have success rates of less than 50%. The trading strategies are built on model assembling. The ensemble assuming that five models produce identical signals (Ensemble 5) achieves the best performance for ethereum and litecoin, with annualized Sharpe ratios of 80.17% and 91.35% and annualized returns (after proportional round-trip trading costs of 0.5%) of 9.62% and 5.73%, respectively. These positive results support the claim that machine learning provides robust techniques for exploring the predictability of cryptocurrencies and for devising profitable trading strategies in these markets, even under adverse market conditions.

## Introduction

Since its inception, coinciding with the international crisis of 2008 and the associated lack of confidence in the financial system, bitcoin has gained an important place in the international financial landscape, attracting extensive media coverage, as well as the attention of regulators, government institutions, institutional and individual investors, academia, and the public in general. For instance, “What is bitcoin?” was the most popular Google search question in the United States and the United Kingdom in 2018 (Marsh 2018). Another example is the launch of bitcoin futures contracts in December 2017 by the Chicago Board Options Exchange (CBOE) and the Chicago Mercantile Exchange, which is indicative of the traditional financial industry’s attempt not to distance itself from this market trend.

The success of bitcoin, measured by its rapid market capitalization growth and price appreciation, led to the emergence of a large number of other cryptocurrencies (e.g., altcoins) that most of the time differ from bitcoin in just a few parameters (e.g., block time, currency supply, and issuance scheme). By now, the market of cryptocurrencies has become one of the largest unregulated markets in the world (Foley et al. 2019), totaling, as of July 2020, more than 5.7 thousand cryptocurrencies, 23 thousand online exchanges and an overall market capitalization that surpasses 270 billion USD (data obtained from the CoinMarketCap site—https://coinmarketcap.com/).

Although initially designed to be a peer-to-peer electronic medium of payment (Nakamoto 2008), bitcoin, and other cryptocurrencies created afterward, rapidly gained the reputation of being pure speculative assets. Their prices are mostly idiosyncratic, as they are mainly driven by behavioral factors and are uncorrelated with the major classes of financial assets; nevertheless, their informational efficiency is still under debate. Consequently, many hedge funds and asset managers began to include cryptocurrencies in their portfolios, while the academic community spent considerable efforts in researching cryptocurrency trading, with emphasis on machine learning (ML) algorithms (Fang et al. 2020). This study examines the predictability and profitability of three major cryptocurrencies—bitcoin, ethereum, and litecoin—using ML techniques; hence, it contributes to this recent stream of literature on cryptocurrencies. These three cryptocurrencies were chosen due to their age, common features, and importance in terms of media coverage, trading volume, and market capitalization (according to CoinMarketCap, together, these three cryptocurrencies represent currently about 75% of the total market capitalization of all types of cryptocurrencies).

Bitcoin as a peer-to-peer (P2P) virtual currency was initially successful because it solves the double-spending problem with its cryptography-based technology that removes the need for a trusted third party. Blockchain is the key technology behind bitcoin, which works as a public (*permissionless*) digital ledger, where transactions among users are recorded. Since no central authority exists, this ledger is replicable among participants (nodes) of the network, who collaboratively maintain it using dedicated software (Yaga et al. 2019). The bitcoin “*ecosystem*” has several features: it is immaterial (being an electronic system that is based on cryptographic entities without any physical representation or intrinsic value), decentralized (does not need a trusted third-party intermediary), accessible and consensual (is open source, with the network managing the balances and transfers of bitcoins), integer (solves the double-spending problem), transparent (information on all transactions is public knowledge), global (has no geographic or economic barriers to its use), fast (confirming a bitcoin trade takes less time than it usually takes to do a normal bank transfer), cheap (transfer costs are relatively low), irreversible and immutable (bitcoin transactions cannot be reversed and once recorded into the blockchain, the trade cannot be modified), divisible (the smallest unit of a bitcoin is called a satoshi, i.e. 10^−8^ of a bitcoin), resilient (the network has been proven to be robust to attacks), pseudonymous (the system does not disclose the identity of users but discloses the addresses of their wallets), and bitcoin supply is capped at 21 million units.

Litecoin and ethereum were launched on October 2011 and August 2015, respectively. Litecoin has the same protocol as bitcoin, and has a supply capped at 84 million units. It was designed to save on the computing power required for the mining process so as to increase the overall processing speed, and to conduct transactions significantly faster, which is a particularly attractive feature in time-critical situations. Ethereum is also a P2P network but unlike bitcoin and litecoin, its cryptocurrency token, called Ether (in the finance literature this token is usually referred to as ethereum), has no maximum supply. Additionally, the ethereum protocol provides a platform that enables applications on its public blockchain such that any user can use it as a decentralized ledger. More specifically, it facilitates online contractual agreement applications (smart contracts) with minimal possibility of downtime, censorship, fraud, or third-party interference. These characteristics help explain the interest that ethereum has gathered since its inception, making it the second most important cryptocurrency.

The main purpose of this study is not to provide a new or improved ML method, compare several competing ML methods, nor study the predictive power of the variables in the input set. Instead, the main objective is to see if the profitability of ML-based trading strategies, commonly evidenced in the empirical literature, holds not only for bitcoin but also for ethereum and litecoin, even when market conditions change and within a more realistic framework where trading costs are included and no short selling is allowed. Other studies have already partly addressed these issues; however, the originality of our paper comes from the combination of all these features, that is, from an overall analysis framework. Additionally, we support our conclusions by conducting a statistical and economic analysis of the trading strategies.

For clarity, we use the term “market conditions” as in Fang et al. (2020), according to whom market conditions are related to bubbles, crashes, and extreme conditions, which are of particular importance to cryptocurrencies. Stated differently, changing market conditions means alternating between periods characterized by a strong bullish market, where most returns are in the upper-tail of the distribution, and periods of strong bearish markets, where most returns are in the lower-tail of the distribution (see, e.g., Balcilar et al. 2017; Zhang et al. 2020).

The remainder of the paper is structured as follows. “Literature review” section provides a literature review, mainly focusing on applications of ML techniques to the cryptocurrencies market. “Data and preliminary analysis” section presents the data used in this study and a preliminary analysis on the price dynamics of bitcoin, ethereum, and litecoin during the period from August 15, 2015 to March 03, 2019. “Methodology” section presents the methodological design focusing on the models used to forecast the cryptocurrencies’ returns and to construct the trading strategies. “Results” section provides the main forecasting and trading performance results. “Conclusions” section presents the conclusions.

## Literature review

Early research on bitcoin debated if it was in fact another type of currency or a pure speculative asset, with the majority of the authors supporting this last view on the grounds of its high volatility, extreme short-run returns, and bubble-like price behavior (see e.g., Yermack 2015; Dwyer 2015; Cheung et al. 2015; Cheah and Fry 2015). This claim has been shifted to other well-implemented cryptocurrencies such as ethereum, litecoin, and ripple (see e.g., Gkillas and Katsiampa 2018; Catania et al. 2018; Corbet et al. 2018a; Charfeddine and Mauchi 2019). The opinion that cryptocurrencies are pure speculative assets without any intrinsic value led to an investigation on the possible relationships with macroeconomic and financial variables, and on other price determinants in the investor’s behavioral sphere. These determinants have been shown to be highly important even for more traditional markets. For instance, Wen et al. (2019) highlight that Chinese firms with higher retail investor attention tend to have a lower stock price crash risk.

Kristoufek (2013) highlights the existence of a high correlation between search queries in Google Trends and Wikipedia and bitcoin prices. Kristoufek (2015) reinforces the previous findings and does not find any important correlation with fundamental variables such as the Financial Stress Index and the gold price in Swiss francs. Bouoiyour and Selmi (2015) study the relationship between bitcoin prices and several variables, such as the market price of gold, Google searches, and the velocity of bitcoin, and find that only lagged Google searches have a significant impact at the 1% level. Polasik et al. (2015) show that bitcoin price formation is mainly driven by news volume, news sentiment, and the number of traded bitcoins. Panagiotidis et al. (2018) examine twenty-one potential drivers of bitcoin returns and conclude that search intensity (measured by Google Trends) is one of the most important ones. In a more recent article, Panagiotidis et al. (2019) find a reduced impact of Internet search intensity on bitcoin prices, while gold shocks seem to have a robust positive impact on these prices. Ciaian et al. (2016) find that market forces and investor attractiveness are the main drivers of bitcoin prices, and there is no evidence that macro-financial variables have any impact in the long run. Zhu et al. (2017) show that economic factors, such as Consumer Price Index (CPI), Dow Jones Industrial Average, federal funds rate, gold price, and most especially the U.S. dollar index, influence monthly bitcoin prices. Li and Wang (2017) find that in early market stages, bitcoin prices were driven by speculative investment and deviated from economic fundamentals. As the market matured, the price dynamics followed more closely the changes in economic factors, such as U.S. money supply, gross domestic product, inflation, and interest rates. Dastgir et al. (2019) observe that a bi-directional causal relationship between bitcoin attention (measured by Google Trends) and its returns exists in the tails of the distribution. Baur et al. (2018) find that bitcoin is uncorrelated with traditional asset classes such as stocks, bonds, exchange rates and commodities, both in normal times and in periods of financial turmoil. Bouri et al. (2017) also document a weak connection between bitcoin and other fundamental financial variables, such as major world stock indices, bonds, oil, gold, the general commodity index, and the U.S. dollar index. Pyo and Lee (2019) find no relationship between bitcoin prices and announcements on employment rate, Producer Price Index, and CPI in the United States; however, their results suggest that bitcoin reacts to announcements of the Federal Open Market Committee on U.S. monetary policy.

That bitcoin prices are mainly driven by public recognition, as Li and Wang (2017) call it—measured by social media news, Google searches, Wikipedia views, Tweets, or comments in Facebook or specialized forums—was also investigated in the case of other cryptocurrencies. For instance, Kim et al. (2016) consider user comments and replies in online cryptocurrency communities to predict changes in the daily prices and transactions of bitcoin, ethereum, and ripple, with positive results, especially for bitcoin. Phillips and Gorse (2017) use hidden Markov models based on online social media indicators to devise successful trading strategies on several cryptocurrencies. Corbet et al. (2018b) find that bitcoin, ripple, and litecoin are unrelated to several economic and financial variables in the time and frequency domains. Sovbetov (2018) shows that factors such as market beta, trading volume, volatility, and attractiveness influence the weekly prices of bitcoin, ethereum, dash, litecoin, and monero. Phillips and Gorse (2018) investigate if the relationships between online and social media factors and the prices of bitcoin, ethereum, litecoin, and monero depend on the market regime; they find that medium-term positive correlations strengthen significantly during bubble-like regimes, while short-term relationships appear to be caused by particular market events, such as hacks or security breaches. Accordingly, some researchers, such as Stavroyiannis and Babalos (2019), study the hypothesis of non-rational behavior, such as herding, in the cryptocurrencies market. Gurdgiev and O’Loughlin (2020) explore the relationship between the price dynamics of 10 cryptocurrencies and proxies for fear (VIX index), uncertainty (U.S. Equity Market Uncertainty index), investors’ sentiment toward cryptocurrencies (measured based on investors’ opinions expressed in a bitcoin forum) and investor perceptions of bullishness/bearishness in the overall financial markets (measured by CBOE put-call ratio). They highlight that investor sentiment is a good predictor of the price direction of cryptocurrencies and that cryptocurrencies can be used as a hedge during times of uncertainty; but during times of fear, they do not act as a suitable safe haven against equities. The results indicate the presence of herding biases among investors of crypto assets and suggest that anchoring and recency biases, if present, are non-linear and environment-specific. In the same line, Chen et al. (2020a) analyze the influence of fear sentiment on bitcoin prices and show that an increase in coronavirus fear has led to negative returns and high trading volume. The authors conclude that during times of market distress (e.g., during the coronavirus pandemic), bitcoin acts more like other financial assets do—it does not serve as a safe haven. In another related strand of literature, several authors have directly studied the market efficiency of cryptocurrencies, especially bitcoin. With different methodologies, Urquhart (2016) and Bariviera (2017) claim that bitcoin is inefficient, while Nadarajah and Chu (2017) and Tiwari et al. (2018) argue in the opposite direction. However, Urquhart (2016) and Bariviera (2017) also point out that after an initial transitory phase, as the market started to mature, bitcoin has been moving toward efficiency.

In the last three years, there has been an increasing interest on forecasting and profiting from cryptocurrencies with ML techniques. Table 1 summarizes several of those papers, presented in chronological order since the work of Madan et al. (2015), which, to the best of our knowledge, is one of the first works to address this issue. We do not intend to provide a complete list of papers for this strand of literature; instead, our aim is to contextualize our research and to highlight its main contributions. For a comprehensive survey on cryptocurrency trading and many more references on ML trading, see, for example, Fang et al. (2020).

**Table 1:** List of studies on machine learning applied to cryptocurrencies prices (organized by chronological and alphabetical order)

Article	Dependent variable	Frequency	Sample period	Models	Type (classification/regression)	Trading strategies (positions/trading costs)	Input set	Main findings
Madan et al. (2015)	Bitcoin prices in USD from Coinbase	10-s, 10-min	5 years since the inception of Bitcoin	Binomial logistic regressions (BLR) and random forest (RF)	Classification	–	Prices and 16 blockchain features	10-min data give a better sensitivity and specificity ratio than the 10-s data
Kim et al. (2016)	Bitcoin, ethereum and ripple prices	Daily	Bitcoin: Dec-2013 to Feb-2016 Ethereum: Aug-2015 to Feb-2016 Ripple: Sept-2015 to Jan-2016	Averaged one-dependence estimators (AODE)	Classification	Long/no trading costs	Trading information, and comments and replies posted in online communities	Comments and replies are good predictors of Bitcoin prices
Żbikowski (2016)	Bitcoin prices in USD from Bitstamp	15-min	Jan-2015 to Feb-2015	Exponential moving average (EMA), box support vector machine (SVM) and volume weighted SVM (VW-SVM)	Classification	Long and short/trading costs of 0.2%	10 technical analysis indicators	VW-SVM is the best model in terms of average return and maximum drawdown
Jiang and Liang (2017)	Prices in USD of the 12 most traded cryptocurrencies at Poloniex	30-min	Jun-2015 to Aug-2016	Convolutional neural networks (CNN) with deep reinforcement learning	Regression	Long and short/trading costs of 0.25%	Returns	Mixed results between CNN portfolio and Online Newton Step and Passive Aggressive Mean Reversion portfolios
Jang and Lee (2018)	Bitcoin price index in USD	Daily	Sep-2011 to Aug-2017	Bayesian neural networks (BNN), linear regression and support vector regressions (SVM)	Regression	–	26 blockchain features, trading information, exchange rates and macroeconomic variables	The BNN is the best prediction model
McNally et al. (2018)	Bitcoin prices in USD from CoinDesk	Daily	Aug-2013 to July-2016	Bayesian recurrent neural (RNN) and long short term memory (LSTM)	Classification and Regression	–	OHLC prices, difficulty, and hash rate of blockchain	The best time lengths are 100 days for the LSTM and 20 days for the RNN
Nakano et al. (2018)	Bitcoin returns in USD from Poloniex	15-min	July- 2016 to Jan-2018	Artificial neural networks (ANN)	Classification	Long, and long and short/transaction costs of 0.025%,0.05% and 0.1%	Returns and 4 technical analysis indicators	Higher performance of the ANN strategy, except in the last month of data. Results are highly sensitive to the model specification and input data
Vo and Yost-Bremm (2018)	Bitcoin prices in USD, CNY, JPY, EUR from 6 online exchanges	1-min	Jan-2012 to Oct-2017	Random forests (RF) and a deep learning model	Classification	Long and short/no trading costs	5 technical analysis indicators	RF is the best model for a frequency of 15-min
Alessandretti et al. (2019)	Price indexes of 1681 cryptocurrencies in USD	Daily	Nov-2015 to Apr-2018	Ensemble of regression trees built by XGboost and long short term memory network	Regression	Long/transaction costs of 0,1%, 0,2%, 0,5% and 1%	Price, market capitalization, market share, rank, volume, and age	All strategies, produce a significant profit (expressed in bitcoin) even with transaction fees up to 0.2%
Atsalakis et al. (2019)	Bitcoin ethereum, litecoin and ripple returns	Daily	Sep-2011 to Oct-2017	PATSOS—a hybrid neuro-fuzzy model	Classification and regression	Long and short/no transaction costs	Returns and prices	PATSOS outperforms other competing methods and produces a return significantly higher than the Buy-and-Hold (B&H) strategy
Catania et al. (2019)	Bitcoin, ethereum, litecoin and ripple returns in USD	Daily	Aug-2015 to Dec-2017	Linear univariate and multivariate regression models, and selections and combinations of those models	Regression	–	Returns and several exogenous financial variables	Statistically significant improvements in forecasting returns when using combinations of univariate models
de Souza et al. (2019)	Bitcoin prices in USD	Daily	May-2012 to May-2017	Artificial neural network (ANN) and support vector machine (SVM)	Classification	Long and short/5 USD	OHLC prices	SVM provides conservative returns on the risk adjusted basis, and ANN generates abnormal profits during short run bull trends
Han et al. (2019)	Bitcoin returns in USD	Daily	April-2013 to Mar-2018	NARX Neural Network	Regression	–	Returns	NARX is effective in predicting the tendency but not the jumps
Huang et al. (2019)	Bitcoin returns in USD	Daily	Jan-2012 to Dec-2017	Trees	Classification	Long and short/no trading costs	124 technical indicators computed from the OHLC prices	Lower volatility, higher win-to-loss ratio and information ratio than those of every simple cut-off strategy or the B&H strategy
Ji et al. (2019b)	Bitcoin returns in USD from Bitstamp	Daily	Nov.-2011 to Dec.-2018	Deep Neural Network (DNN), Long Short Term Memory (LSTM), Convolutional Neural Network (CNN), Deep Residual Network (ResNet), combination of CNNs and RNNs (CRNN) and their combinations	Classification and regression	Long/no transaction costs	Prices and 17 blockchain features	Performances of the prediction models were comparable, LSTM is the best prediction model, DNN models are the best classification models, classification models were more effective for trading
Lahmiri and Bekiros (2019)	Bitcoin, digital cash and ripple prices in USD	Daily	Bitcoin: July-2010 to Oct-2018 Digital Cash: Feb-2010 to Oct-2018 Ripple: Jan-2015 to Oct-2018	Long Short Term Memory (LSTM) and Generalized Regression Neural Networks (GRNN)	Regression	–	Prices	Predictability of LSTM is significantly higher than of GRNN
Mallqui and Fernandes (2019)	Bitcoin prices in USD	Daily	Apr-2013 to Apr-2017	Artificial neural networks (ANN), support vector machine (SVM) and ensembles	Classification and Regression	–	OHLC prices, Blockchain information and several exogenous financial variables	Ensemble of recurrent neural networks and a Tree classifier is the best classification model, while SVM is the best regression model
Shintate and Pichl (2019)	Bitcoin returns in CNY and USD from OkCoin	1-min	Jun-2013 to Mar-2017	Random sampling method (RSM)	Classification	Long and short/No transaction costs	OHLC prices	The proposed RSM outperforms several alternatives, but the profit rates do not exceed those of the B&H strategy
Smuts (2019)	Bitcoin and ethereum prices in USD	1-h	Dec-2017 to Jun-2018	Long short term memory recurrent neural network (LSTM)	Classification	–	Prices, volumes, Google trends, and Telegram chat groups dedicated to bitcoin and ethereum trading	Telegram data is a better predictor of bitcoin, while GoThe ensemble, by unweighted average of the four trading signals from the four models, after resampling the data, gives the best results.ogle Trends is a better predictor of ethereum, especially in one-week period
Borges and Neves (2020)	Prices from Binance 100 cryptocurrencies pairs with the most traded volume in USD	1-min	For each pair since beginning of trading at Binance until oct-2018	Logistic regression, random forest, support vector machine, and gradient tree boosting and an ensemble of these models	Classification	Long/transaction costs of 0.1%	Returns, resampled returns, and 11 technical indicators	
Chen et al. (2020b)	Bitcoin price index and trading prices from Binance in USD	5-min and daily	July-2017 to Jan-2018 for 5-min and Feb-2017, to Feb-2019 for daily	Logistic Regression (LR), Linear Discriminant Analysis (LDA), Random Forest (RF), XGBoost (XGB), Support Vector Machine (SVM), and Long Short-Term Memory (LSTM)	Classification	–	5-min: OHLC prices and trading volume. Daily: 4 Blockchain features, 8 marketing and trading variables, Google trend search volume index, Baidu media search volume, and gold spot price	For 5-min data machine learning models achieved better accuracy than LR and LDA, with LSTM achieving the best result (67% accuracy). For daily data, LR and LDA are better, with an average accuracy of 65%
Chu et al. (2020)	Bitcoin, ethereum, dash, litecoin, MaidSafeCoin, monero and ripple from CryptoCompare in USD	Hourly	Feb-2017 to Aug-2017	Exponential Moving Averages (EMA) for time series and cross-sectional portfolios	Classification and Regression	Long and short/No transaction costs	Trading prices	Momentum trading does not beat the passive trading strategies
Sun et al. (2020)	42 cryptocurrencies	Daily	Jan-2018 to Jun-2018	LightGBM, SVM support vector machines (SVM) and Random Forests (RF)	Classification	–	Trading data and macroeconomic variables	LightGBM outperforms SVM and RF, and the accuracy is higher for 2 weeks predictions

In a nutshell, all these papers point out that independent of the period under analysis, data frequency, investment horizon, input set, type (classification or regression), and method, ML models present high levels of accuracy and improve the predictability of prices and returns of cryptocurrencies, outperforming competing models such as autoregressive integrated moving averages and Exponential Moving Average. Around half of the surveyed studies also compare the performance of the trading strategies devised upon these ML models and against the passive buy-and-hold (B&H) strategy (with and without trading costs). In the competition between different ML models there is no unambiguous winner; however, the consensual conclusion is that ML-based strategies are better in terms of overall cumulative return, volatility, and Sharpe ratio than the passive strategy. However, most of these studies analyze only bitcoin, cover a period of steady upward price trend, and do not consider trading costs and short-selling restrictions.

From the list in Table 1, studies that are closer to the research conducted here are Ji et al. (2019b), where the main goal is to compare several ML techniques, and Borges and Neves (2020), where the main goal is to show that assembling ML algorithms with different data resampling methods generate profitable trading strategies in the cryptocurrency markets. The main differences between our research and the first paper are that we consider not only bitcoin but also, ethereum and litecoin, and we also consider trading costs. Meanwhile, the main differences with the second paper are that we study daily returns and use blockchain features in the input set instead of one-minute returns and technical indicators.

## Data and preliminary analysis

The daily data, totaling 1,305 observations, on three major cryptocurrencies—bitcoin, ethereum, and litecoin—for the period from August 07, 2015 to March 03, 2019 come from two sources. The sample begins one week after the inception of ethereum, the youngest of the three cryptocurrencies. Exchange trading information—the closing prices (the last reported prices before 00:00:00 UTC of the next day) and the high and low prices during the last 24 h, the daily trading volume, and market capitalization—come from the CoinMarketCap site. These variables are denominated in U.S. dollars. Arguably these last two trading variables, especially volume, may help the forecasting returns (see for instance Balcilar et al. 2017). Additionally, blockchain information on 12 variables, also time-stamped at 00:00:00 UTC of the next day, come from the Coin Metrics site (https://coinmetrics.io/).

For each cryptocurrency, the dependent variables are the daily log returns, computed using the closing prices or the sign of these log returns. The overall input set is formed by 50 variables, most of them coming from the raw data after some transformation. This set includes the log returns of the three cryptocurrencies lagged one to seven days earlier (the returns of cryptocurrencies are highly interdependent at different frequencies, as shown in Bação et al. 2018; Omane-Adjepong and Alagidede 2019; and Hyun et al. 2019) and two proxies for the daily volatility, namely the relative price range, $RR_{t}$, and the range volatility estimator of Parkinson (1980), $\sigma_{t}$, computed respectively as:

1 $$RR_{t} = 2\frac{{H_{t} - L_{t} }}{{H_{t} + L_{t} }},$$

2 $$\sigma_{t} = \sqrt {\frac{{\left( {\ln \left( {H_{t} /L_{t} } \right)} \right)^{2} }}{4\ln \left( 2 \right)}} ,$$

where $H_{t}$ and $L_{t}$ are the highest and lowest prices recorded at day *t*. More precisely, the set includes the first lag of $RR_{t}$ and lags one to seven of $\sigma_{t}$ (for other applications of the Parkinson estimator to cryptocurrencies see, for example, Sebastião et al. 2017 and Koutmos 2018).

The first lag of the other exchange trading information and network information of the corresponding cryptocurrency are included in the input set, except if they fail to reject the null hypothesis of a unit root of the augmented Dickey–Fuller (ADF) test, in which case we use the lagged first difference of the variable. This differencing transformation is performed on seven variables. The data set also includes seven deterministic day dummies, as it seems that the price dynamics of cryptocurrencies, especially bitcoin, may depend on the day of the week, (Dorfleitner and Lung 2018; Aharon and Qadan 2019; Caporale and Plastun 2019). Table 2 presents the input set used in our ML experiments.

**Table 2:** Initial input set for each cryptocurrency

Variable	Description	Diff.	Lags
*Exchange trading information*			
Returns	Logarithmic returns of bitcoin, ethereum and litecoin computed using closing prices in USD. These prices are volume weighted average prices (VWAP) considering the most important online exchanges	No	1–7
Volume	Trading volume, denominated in USD, in the most important online exchanges	No	1
Capitalization	Market capitalization, denominated in USD, computed as the multiplication of the reference price by a rough estimation of the number of circulating units of each cryptocurrency	Yes	1
Relative price change	Proxy for daily volatility, computed using daily high and low prices in USD (Eq. 1)	No	1
Parkinson’s volatility	Proxy for daily volatility, computed using daily high and low prices in USD (Eq. 2)	No	1–7
*Blockchain information*			
On-chain volume	Total value of outputs on the blockchain, denominated in USD	No	1
Adjusted on-chain volume	On-chain transaction volume after heuristically filtering the non-meaningful economic transactions	No	1
Median value	Median value in USD per transaction	No	1
Number of transactions	Number of transactions on the public blockchain	Yes	1
New coins	Number of new coins created	No	1
Total fees	Total fees payed to use the network in native currency	No	1
Median fees	Median fee in native currency per transaction	No	1
Active addresses	Number of unique active addresses in the network	Yes	1
Average difficulty	Mean difficulty of finding a hash that meets the protocol-designated requirement, i.e., the difficulty of finding a new block	Yes	1
Number of blocks	Number of new blocks that were included in the main chain	Yes	1
Block size	Mean size in bytes of all blocks	Yes	1
Number of payments	Number of recipients of the transactions, which can be more than one per transaction given the possibility of payment batching	Yes	1
*Deterministic variables*			
Daily dummies	Seven daily dummies. One for each day-of-the-week	No	-

This table lists the input set for each cryptocurrency, composed of 50 variables: 31 obtained from exchange trading information, 12 obtained from blockchain information, and 7 day-of-the-week dummies. All these variables have a daily frequency time-stamped at 00:00:00 UTC of the next day. The column “Diff” indicates if the variable was used in levels or was differentiated

**Fig. 1:**
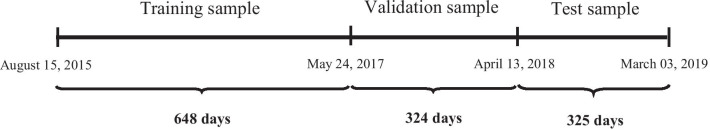
Data partition of the sample into training, validation and test sub-samples, according to the rule 50–25–25%

In this work, we use the three-sub-samples logic that is common in ML applications with a rolling window approach. The first 648 days (about 50% of the sample) are used just for training purposes; thus, we call this set of observations as the “training sample.” From day 649 to day 972 (324 days, about 25% of the sample), each return is forecasted using information from the previous 648 days. The performance of the forecasts obtained in these observations is used to choose the set of variables and hyperparameters. This set of observations is not exactly the validation sub-sample used in ML, since most observations are used both for training and for validation purposes (e.g., observation 700 is forecasted using the 648 previous observations, and it is also used to forecast the returns of the following days). Despite not being exactly the validation sub-sample, as usually understood in ML, it is close to it, since the returns in this sub-sample are the ones that are compared to the respective forecast for the purpose of choosing the set of variables and hyperparameters. Thus, for the sake of simplicity, we call this set of returns the “validation sample”. From day 973 to day 1297 (325 days, about 25% of the sample), each return is forecasted using information from the previous 648 days, applying the models that showed the best performance in forecasting the returns in the “validation sample”. Therefore, as in the case of our “validation sample”, this set does not exactly correspond to the test sample as understood in ML. However, it is close to it since it is used to assess the quality of the models in new data. We refer to it as the “test sample”. Fig. 1 presents the sample partition.

**Fig. 2:**
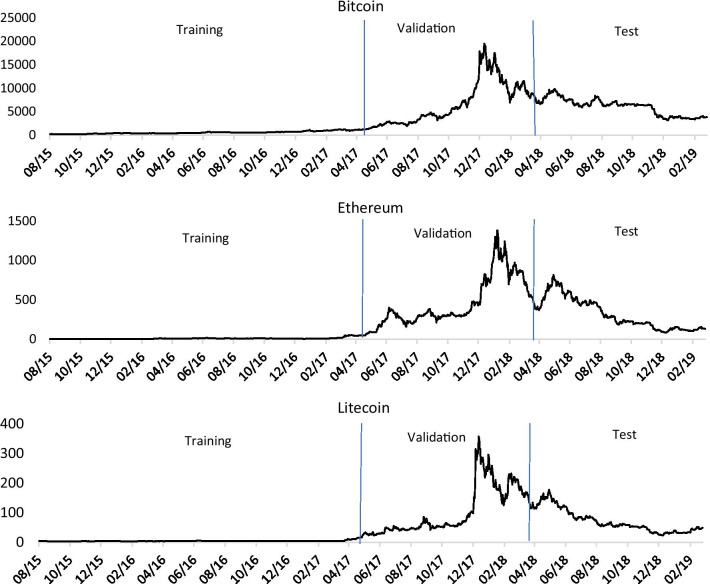
The daily closing volume weighted average prices of bitcoin, ethereum, and litecoin for the period from August 15, 2015 to March 3, 2019 come from the CoinMarketCap site. The figure shows the partition of the sample into training, validation, and test sub-samples, according to the 50–25–25% rule

The price paths of the three cryptocurrencies are shown in Fig. 2, which indicates that the price dynamics of the three cryptocurrencies are quite different across the three sub-samples. Although at first glance, looking at Fig. 2, it seems that the prices are smoother in the training sample than in the latter periods, this is in fact an illusion, caused by the lower levels of prices in the first period. Then, in the first half of the validation sample, the prices show an explosive behavior, followed in the second half by a sudden and sharp decay. In the test sample there is an initial month of an upward movement and then a markedly negative trend. Roughly speaking, at the end of the test sample, the prices are about double the prices in the beginning of the validation sample.

Table 3 presents some descriptive statistics of the log returns of the three cryptocurrencies. All cryptocurrencies have a positive mean return in the training and validation sub-samples, and a negative mean return in the test sample; however, only the mean returns of bitcoin and ethereum in the training samples are significant at the 1% and 5% levels, respectively. During the overall sample period, from August 15, 2015 to March 03, 2019, the daily mean returns are 0.21% (significant at 10%), 0.33%, and 0.19% for bitcoin, ethereum, and litecoin, respectively. The median returns are quite different across the three cryptocurrencies and the three subsamples. The bitcoin median returns are positive in the three subsamples, the ethereum median return is only positive in the validation sub-sample (resulting in a median return of − 0.12% in the overall period), while litecoin shows a negative median return in the last two sub-samples and a zero median return in the first sub-sample (resulting in a zero median in the overall period).

**Table 3:** Summary statistics on the returns of bitcoin, ethereum and litecoin

	1st Sub-sample (training) 15-Aug-2015 to 23-May-2017 (648 obs.)	2nd Sub-sample (validation) 24-May-2017 to 12-Apr-2018 (324 obs.)	3rd sub-sample (test) 13-Apr-2018 to 03-Mar-2019 (325 obs.)	Overall sample 15-Aug-2015 to 03-Mar-2019 (1297 obs.)
*Bitcoin*				
Mean (%)	0.3345***	0.3777	− 0.2210	0.2061*
Median (%)	0.2676	0.6255	0.0850	0.2294
Min. (%)	− 20.06	− 20.75	− 14.36	− 20.75
Max. (%)	11.29	22.51	10.82	22.51
SD (%)	3.111	5.700	3.271	3.958
Skewness	− 1.149	0.0571	− 0.4784	− 0.2615
Exc. kurtosis	7.807	1.743	2.635	4.807
*ρ*(1)	0.0004	0.0224	− 0.0752	0.0041
*Ethereum*				
Mean (%)	0.7098**	0.3076	− 0.4048	0.3300
Median (%)	− 0.1469	0.0737	− 0.2401	− 0.1237
Min. (%)	− 31.55	− 25.89	− 20.69	− 31.55
Max. (%)	30.28	23.47	16.61	30.28
SD (%)	7.164	6.686	5.142	6.602
Skewness	0.2979	0.0443	− 0.3636	0.2066
Exc. kurtosis	3.697	1.662	2.034	3.401
*ρ*(1)	0.0688*	0.0263	− 0.0681	0.0418
*Litecoin*				
Mean (%)	0.3202	0.4300	− 0.3025	0.1916
Median (%)	0.0000	− 0.0109	− 0.3210	0.0000
Min. (%)	− 20.92	− 39.52	− 14.72	− 39.52
Max. (%)	51.04	38.93	26.87	51.04
SD (%)	4.659	8.052	4.872	5.746
Skewness	2.965	0.5794	0.2720	1.264
Exc. kurtosis	28.72	4.880	3.263	12.34
*ρ*(1)	0.0184	0.0297	− 0.0719	0.0131

This table shows some descriptive statistics of the log-returns of bitcoin, ethereum and litecoin. The values for the first five statistics are presented in percentage. The significance of the mean return is assessed using the t-statistic with Newey–West HAC standard error, with a Bartlett kernel bandwidth of 8. *ρ*(1) is the first order autocorrelation. Significance at the 10%, 5% and 1% levels are denoted by *, ** and ***, respectively

As already documented in the literature, these cryptocurrencies are highly volatile. This is evident from the relatively high standard deviations and the range length. The standard deviations range from 3.11% for bitcoin in the training period to 8.05% for litecoin in the validation period, meaning that the volatility is higher than the mean by at least a factor of 10 for all cryptocurrencies. All the minima and maxima achieve two-digit percent returns, with the amplitude between the extreme values higher for litecoin, and the minimum (maximum) log return at − 39.52% (51.04%). It is noteworthy that the dynamics of the volatility of ethereum, which is decreasing through the three periods, is different from that of the other two cryptocurrencies. Specifically, for bitcoin and litecoin, the volatility increases from the first to the second subsamples and decreases afterwards, reaching slightly higher values than in the training sample. Overall, bitcoin is the least volatile among the three cryptocurrencies.

The skewness is negative in the first and third sub-samples for bitcoin and in the third sub-sample for ethereum. In the overall sample, only bitcoin presents a negative skewness (− 0.26), while the skewness of litecoin reaches the value of 1.26. All cryptocurrencies present excess kurtosis, especially during the training sub-sample.

The daily first-order autocorrelations are all positive in the first and second sub-samples and negative in the last one; however, only the autocorrelation of ethereum during the training sample, which assumes a value of 6.88%, is significant (at the 10% level). Overall, the autocorrelation coefficients are quite low, at 0.41%, 4.18%, and 1.31% for bitcoin, ethereum and litecoin, respectively. This implies that most of the time the daily returns do not have significant information that can be used to preview linearly the returns for the next day.

A comparison of the previous statistics between sub-samples reveals several features. First, the training period is characterized by a steady upward price trend although the volatility of returns is not substantially lower than in the latter periods, and in fact, for ethereum, the volatility in this initial period is higher than afterward. Second, although during the validation period, cryptocurrencies experience an explosive behavior—followed by a visible crash—the mean returns are still positive. Third, the test period differs from the previous periods mainly by its negative mean return and negative first-order autocorrelation, which indicates that the negative price trend that started at the end of 2017 prevailed in this last sub-sample.

## Methodology

This study examines the predictability of the returns of major cryptocurrencies and the profitability of trading strategies supported by ML techniques. The framework considers several classes of models, namely, linear models, random forests (RFs), and support vector machines (SVMs). These models are used not only to produce forecasts of the dependent variable, which is the returns of the cryptocurrencies (regression models), but also to produce binary buy or sell trading signals (classification models).

Random forests (RFs) are combinations of regression or classification trees. In this application, regression RFs are used when the goal is to forecast the next return, and classification RFs are used when the goal is to get a binary signal that predicts whether the price will increase or decrease the next day. The basic block of RFs is a regression or a classification tree, which is a simple model based on the recursive partition of the space defined by the independent variables into smaller regions. In making a prediction, the tree is thus read from the first node (the root node); the successive tests are made; and successive branches are chosen until a terminal node (the leaf node) is reached, which defines the value to be predicted for the dependent variable (the forecast for the next return or the binary signal that predicts whether the price is going to increase or decrease the next day). An RF uses several trees. In each tree node, a random subset of the independent variables and that of the observations in the training dataset are used to define the test that leads to choosing a branch. RF forecasts are then obtained by averaging the forecasts made by the different trees that compose it (in the case of a regression RF), or by choosing the binary signal chosen by the largest number of trees (in the case of a classification RF).

SVMs can also be used for classification or regression tasks. In the case of binary classification, SVMs try to find the hyperplane that separates the two outputs that leave the largest margin, defined as the summation of the shortest distance to the nearest data point of both categories (Yu and Kim 2012). Classification errors may be allowed by introducing slack variables that measure the degree of misclassification and a parameter that determines the trade-off between the margin size and the amount of error. In the case of regression SVMs, the objective is to find a model that estimates the values of the output to avoid errors that are larger than a pre-defined value $\left( \varepsilon \right)$. This means that SVMs use an “$\varepsilon$-insensitive loss function” that ignores errors that are smaller but penalizes errors that are greater than this threshold $\left( \varepsilon \right)$. The coefficients of the model are obtained by minimizing a function that consists of the sum of a function of the reciprocal of the “margin” with a penalty for deviations larger than $\varepsilon$ between the predicted and the original values of the output. Formally, if $\left\{ {\left( {{\varvec{x}}_{1} ,y_{1} } \right), \ldots ,\left( {{\varvec{x}}_{{\varvec{l}}} ,y_{l} } \right)} \right\}$ is the training data (usually normalized, that is, centered and scaled), the SVM regression will estimate a function

3 $$f\left( {\varvec{x}} \right) = \langle{\varvec{w}},{\varvec{x}}\rangle + b,$$

where $\langle{\varvec{w}},{\varvec{x}}\rangle$ denotes the inner product. In the case of a regression SVM, the margin can be seen as the “flatness” of the obtained model (Smola and Schölkopf 2004), that is, the reciprocal of the Euclidean norm of the vector of the coefficients, $1/{\parallel\varvec{w}}\parallel$ (Yu and Kim 2012). Denoting by $C > 0$ the parameter that defines the trade-off between the margin size and the prediction errors larger than $\varepsilon ,$ and by $\xi_{i}$ and $\xi_{i}^{*}$ the slack variables that measure such errors, the estimation of Model (3) may be performed by solving the following equation (Smola and Schölkopf 2004):

4 $$\begin{aligned} & {\text{min }}\frac{1}{2}{\parallel\varvec{w}\parallel}^{2} + C\mathop \sum \limits_{i = 1}^{l} \left( {\xi_{i} + \xi_{i}^{*} } \right) \\ & \quad \left\{ {\begin{array}{*{20}l} {y_{i} - \langle{\varvec{w}},{\varvec{x}}_{{\varvec{i}}}\rangle - b \le \varepsilon + \xi_{i} ,\quad i \in \left\{ {1; \ldots ;l} \right\}} \\ {\langle{\varvec{w}},{\varvec{x}}_{{\varvec{i}}}\rangle + b - y_{i} \le \varepsilon + \xi_{i}^{*} ,\quad i \in \left\{ {1; \ldots ;l} \right\}} \\ {\xi_{i} ,\xi_{i}^{*} \ge 0} \\ \end{array} } \right. \\ \end{aligned}$$

SVM can handle non-linear models by using the “kernel trick.” First, the original data is mapped into a new high-dimensional space, where it is possible to apply linear models for the problem. Such mapping is based on kernel functions, and SVMs operate on the dual representation induced by such functions. SVMs use models that are linear in this new space but non-linear in the original space of the data. Common kernel functions used in SVMs include the Gaussian and the polynomial kernels (Tay and Cao 2001; Ben-Hur and Weston 2010). According to Tay and Cao (2001), Gaussian kernels tend to have good performance under general smoothness assumptions; thus, they are commonly used (e.g., Patel et al. 2015). It is also possible to use the original linear models, usually referred to as a “linear kernel.”

RFs and SVMs are implemented in R, using packages randomForest (Liaw and Wiener 2002) and e1071 (Meyer et al. 2017), respectively. For a reference on the practical application of these methods in R, see Torgo (2016).

In ML applications to time series, the data are commonly split into a training set, used to estimate the different models, a validation set, in which the best in-class model is chosen, and a test set, where the results of the best models are assessed. In this work, the main concerns when defining the different data subsets are: on the one hand, to avoid all risks of data snooping, and on the other hand, to make sure that the results obtained in the test set could be considered representative. The approach is first, to split the dataset into two equal lengths of sub-samples. The first sub-sample is used for training, which means that it is only used to build the initial models by fitting the model parameters to the data. The other half of the data is then partitioned into the validation sub-sample (25% of the data), and into the testing sub-sample (the last 25% of the data). The validation sub-sample is used to choose the best model of each class, and the test sub-sample is used for assessing the forecasting and profitability performance of the models.

For each class, choosing the best model means defining a set of “hyperparameters” and choosing a set of explanatory variables for that method. This analysis uses parameterizations close to the defaults of R or R packages. Table 4 presents the parameters that were tested in the ML experiments and highlights the ones that lead to the best models.

**Table 4:** Parameters tested in the ML models and parameters leading to the best model

Model	Hyperparameters used	Hyperparameters of the model with the best performance in the validation sample
RF	Number of trees: 500, 1000, 1500	Bitcoin: 1500 trees, 50.0% of the variables sampled at each split
	Percentage of variables sampled at each split: 50.0%, 33.3%	Litecoin: 1500 trees, 50.0% of the variables sampled at each split
		Ethereum: 500 trees, 50.0% of the variables sampled at each split
RF-binary	Number of trees: 500, 1000, 1500	Bitcoin: 500 trees, 33.3% of the variables sampled at each split
	Percentage of variables sampled at each split: 50.0%, 33.3%	Litecoin: 1000 trees, 33.3% of the variables sampled at each split
		Ethereum: 1500 trees, 50.0% of the variables sampled at each split
SVM	Kernel: radial, linear, polynomial	Bitcoin: polynomial kernel, gamma = 0.20
	Gamma: 0.05. 0.10, 0.20	Litecoin: radial kernel, gamma = 0.05
		Ethereum: radial kernel, gamma = 0.10
SVM-binary	Kernel: radial, linear, polynomial	Bitcoin: radial kernel, gamma = 0.20
	Gamma: 0.05. 0.10, 0.20	Litecoin: polynomial kernel, gamma = 0.10
		Ethereum: radial kernel, gamma = 0.10

This table presents all combinations of hyperparameters used in the experiments. The hyperparameters of the model with the best performance in the validation sample were then used to define the trading strategies in the test sample. For Random Forests (RF), the remaining hyperparameters were kept at the defaults of the randomForest R package. For Support Vector Machines (SVM), the remaining hyperparameters were kept at the defaults of the e1071 R package

We also tried 18 different sets of input variables that might have a significant influence on the results. Specifically, by always including the day dummies and the first lag of the relative price range, we have tried all lag lengths for the cryptocurrencies vector and for the range volatility estimator from one to seven, with and without other market and blockchain variables (14 sets); and the first lag of the other cryptocurrencies and of the range volatility estimator combined with lags 1–2 and 1–3 of the dependent cryptocurrency, with and without other market and blockchain variables (4 sets).

For each model class, the set of variables and hyperparameters that lead to the best performance is chosen according to the average return per trade during the validation sample, and because the models always prescribe a non-null trading position, these values can also be interpreted as daily averages. The procedure is as follows. For each observation in the validation sample, a model is estimated using the previous 648 observations (the number of observations in the training sub-sample), that is, using a rolling window with a fixed length. For example, the forecast for the first day in the validation sample, day 649 of the overall sample, is obtained using 648 observations from the first day to the last day of the training sample, then the window is moved one day forward to make the forecast for the second day in the validation sample, that is, this forecast is obtained using data from day 2 to day 649, and so on, until all the 324 forecasts are made for the validation period (for day 649 to day 970 of the overall sample). Then, a trading strategy is defined based on the binary signals generated by the model (in the case of classification models), or on the sign of the return forecasts (in the case of regression models). In both types of models, we open/keep a long position if the model forecasts a rise in the price for the next day, and we leave/stay out of the market if the model forecasts a decline in the price for the next day. For classification models, this forecast comes in the form of a binary signal, and for regression models it comes in the form of a return forecast. The trading strategy is used to devise a position in the market at the next day, and its returns are computed and averaged for the overall validation period. Hence, the models, that is, the best sets of input variables, are assessed using a time series of 324 outcomes (the number of observations in the validation sample). The best model of each class, and only this model, is then used in the test set, using a procedure that is similar to the one used in the validation set.

The predictability of the models in the validation and test sub-samples is assessed via several metrics. Besides the success rate that is given by the relative number of times that the model predicts the right signal of the one-day ahead return and can be computed both for the regression and classification models, we also report the mean absolute error (MAE), the root mean square error (RMSE), and Theil’s U^2^. This last metric represents the ratio of the mean square error (MSE) of the proposed model to the MSE of a naïve model that predicts that the next return is equal to the last known one. Hence, when U^2^ is less than unity, this means that the proposed model incorporates a forecasting improvement in relation to the naïve model. Notice that the criterion used to obtain the best in-class model is the maximization of the out-of-sample average return and not the minimization of the forecast errors; hence, forecasting accuracy is not the best way to measure the model’s performance.

Given the results reported in the literature that indicate model averaging or assembling provides good outcomes for the cryptocurrencies’ market (see, e.g., Catania et al. 2019; Mallqui and Fernandes 2019), we proceed with the statistical and economic analysis on the profitability of the trading strategies based on ML considering three model ensembles. Basically, a long position in the market is created if at least four, five, or six individual models (out of the six models) agree on the positive trading signal for the next day. If the threshold number of forecasts in agreement is not met for the next day, the trader does not enter into the market or the existing positive position is closed, and the trader gets out of the market. Notice that the trading strategies only consider the creation of long positions, because short selling in the market of cryptocurrencies may be difficult or even impossible. Model averaging or assembling of basic ML models are quite simple classifier procedures; other more complex classification procedures presented in the literature could be used in this framework, with a high probability of producing better results. For instance, Kou et al. (2012) address the issue of classification algorithm selection as a multiple criteria decision-making (MCDM) problem and propose an approach to resolve disagreements among methods based on Spearman's rank correlation coefficient. Kou et al. (2014) presents an MCDM-based approach to rank a selection of popular clustering algorithms in the domain of financial risk analysis. Li et al. (2020a) use feature engineering to improve the performances of classifiers in identifying malicious URLs, and Li et al. (2020b) propose adaptive hyper-sphere (AdaHS), an adaptive incremental classifier, and its kernelized version: Nys-AdaHS, which are especially suitable for dynamic data in which patterns change.

The assessment of the profitability of the trading strategies is conducted using a battery of performance indicators. The win rate is equal to the ratio between the number of days when the ensemble model gives the right positive sign for the next day and the total of the days in the market. The mean and standard deviation of the returns when the positions are active are also shown. The annual return is the compound return per year given by the accumulated discrete daily returns considering all days in the test sample, including zero-return days when the strategies prescribe not being in the market. The annualized Sharpe ratio is the ratio between the daily return and the standard deviation of daily returns, considering all days in the test sample, multiplied by $\sqrt {365}$. The bootstrap p-values are the probabilities of the daily mean return of the proposed model, and considering all days in the sample, is higher than the daily mean return of the B&H strategy that consists of being long all the time, given the null that these mean returns are equal. The tail risk is measured by the Conditional Value at Risk (CVaR) at 1% and the maximum drawdown. The former measures the average loss conditional upon the Value at Risk (VaR) at the 1% level being exceeded. The latter measures the maximum observed loss from a peak to a trough of the accumulated value of the trading strategy, before a new peak is attained, relative to the value of that peak.

We also present the annual return after considering transaction costs. As highlighted by Alessandretti et al. (2019), in most exchange markets, proportional transaction costs are typically between 0.1 and 0.5%. Thus, even if the investor trades in a high-fee online exchange, it seems that a proportional round-trip transaction cost of 0.5% is a good estimate of the overall trading cost, including explicit and implicit costs such as bid–ask spreads and price impacts. This is a higher figure than is used in most of the related literature.

## Results

Table 5 shows the sets of variables that maximize the average return of a trading strategy in the validation period—without any trading costs or liquidity constraints—devised upon the trading positions obtained from rolling-window, one-step forecasts. These sets are kept constant and then used in the test sample. Several patterns emerge from this table. First, all models use the lag returns of the three cryptocurrencies, the lagged volatility proxies, and the day-of-the-week dummies. Second, in most cases, the lag structure is the same for those variables for which more than one lag is allowed, that is, for returns and Parkinson range volatility estimator. Third, the other trading variables (i.e., the daily trading volume and market capitalization) and network variables are only used in the binary models.

**Table 5:** Sets of variables used in the models

Variables	Returns	Volatility	Other trading variables	Network	Daily dummies	# variables
*Bitcoin*						
Linear	BTC[− 1, − 2]	RR[− 1]	No	No	Yes	14
	ETH[− 1]	$\sigma$[− 1, − 2]				
	LTC[− 1]					
Linear-binary	BTC[− 1, …, − 7]	RR[− 1]	Yes	Yes	Yes	48
	ETH[− 1, …, − 7]	$\sigma$[− 1, …, − 7]				
	LTC[− 1, …, − 7]					
RF	BTC[− 1, − 2]	RR[− 1]	No	No	Yes	16
	ETH[− 1, − 2]	$\sigma$[− 1, − 2]				
	LTC[− 1, − 2]					
RF-binary	BTC[− 1, − 2]	RR[− 1]	No	No	Yes	14
	ETH[− 1]	$\sigma$[− 1, − 2]				
	LTC[− 1]					
SVM	BTC[− 1, − 2]	RR[− 1]	No	No	Yes	16
	ETH[− 1, − 2]	$\sigma$[− 1, − 2]				
	LTC[− 1, − 2]					
SVM-binary	BTC[− 1, − 2]	RR[− 1]	Yes	Yes	Yes	26
	ETH[− 1]	$\sigma$[− 1, − 2]				
	LTC[− 1]					
*Ethereum*						
Linear	ETH[− 1, − 2]	RR[− 1]	No	No	Yes	14
	BTC[− 1]	$\sigma$[− 1, − 2]				
	LTC[− 1]					
Linear-binary	ETH[− 1, − 2, − 3]	RR[− 1]	Yes	Yes	Yes	32
	BTC[− 1, − 2, − 3]	$\sigma$[− 1, − 2, − 3]				
	LTC[− 1, − 2, − 3]					
RF	ETH[− 1, − 2]	RR[− 1]	No	No	Yes	16
	BTC[− 1, − 2]	$\sigma$[− 1, − 2]				
	LTC[− 1, − 2]					
RF-binary	ETH[− 1, − 2]	RR[− 1]	No	No	Yes	16
	BTC[− 1, − 2]	$\sigma$[− 1, − 2]				
	LTC[− 1, − 2]					
SVM	ETH[− 1, …, − 4]	RR[− 1]	No	No	Yes	24
	BTC[− 1, …,-4]	$\sigma$[− 1, …, − 4]				
	LTC[− 1, …,-4]					
SVM-binary	ETH[− 1, …, − 5]	RR[− 1]	No	No	Yes	28
	BTC[− 1, …, − 5]	$\sigma$[− 1, …, − 5]				
	LTC[− 1, …, − 5]					
*Litecoin*						
Linear	LTC[− 1, …, − 6]	RR[− 1]	No	No	Yes	32
	BTC[− 1, …, − 6]	$\sigma$[− 1, …, − 6]				
	ETH[− 1, …, − 6]					
Linear-binary	LTC[− 1, …, − 6]	RR[− 1]	Yes	Yes	Yes	44
	BTC[− 1, …, − 6]	$\sigma$[− 1, …, − 6]				
	ETH[− 1, …, − 6]					
RF	LTC [− 1, − 2]	RR[− 1]	No	No	Yes	16
	BTC[− 1, − 2]	$\sigma$[− 1, − 2]				
	ETH[− 1, − 2]					
RF-binary	LTC [− 1]	RR[− 1]	No	No	Yes	12
	BTC[− 1]	$\sigma$[− 1]				
	RTH [− 1]					
SVM	LTC [− 1, …, − 5]	RR[− 1]	Yes	Yes	Yes	40
	BTC[− 1, …, − 5]	$\sigma$[− 1, …, − 5]				
	ETH[− 1, …, − 5]					
SVM-binary	LTC [− 1, − 2,-3]	RR[− 1]	No	No	Yes	20
	BTC[− 1, − 2,-3]	$\sigma$[− 1, − 2, − 3]				
	ETH[− 1, − 2,-3]					

This table shows the best input sets obtained in the validation sample, i.e. those variables that maximize the 1-step out-of-sample average return, based on a rolling window with a length of 648 days. These sets of variables are then used in the test sample. The second column refers to the lagged returns of the three cryptocurrencies, which could go up to lag 7. The third column refers to two volatility estimators of the dependent cryptocurrency, namely the relative price range, $RR_{t}$, and the range estimator of Parkinson (1980), $\sigma_{t}$. For the first estimator only the first lag is used, while for $\sigma_{t}$ it was considered a maximum lag structure up to lag 7. The number of lags used in these variables are in squared brackets. The fourth column refers to other trading variables, namely the daily trading volume and market capitalization. The fifth column refers to network variables. All the models that include these variables, consider a subset of the initial network variables, however all these subsets do not include the median transaction value and the number of transactions on the public blockchain. The sixth column refers to dummies corresponding to the day-of-the-week.

Table 6 presents the metrics on the forecasting ability of the regression models and the success rate for the binary versions of the linear, RF, and SVM models (classification).

**Table 6:** Forecasting ability of the models

Variables	Success rate (classification)	Success rate (regression)	MAE	RMSE	Theil’s U^2^
*Validation sample*					
Linear (BTC)	49.69	57.72	4.25	5.79	71.49
Linear (ETH)	45.68	48.46	4.97	6.85	92.13
Linear (LTC)	49.38	45.37	5.73	8.14	98.13
RF (BTC)	57.10	56.17	4.30	5.77	93.80
RF (ETH)	50.00	55.86	5.06	6.85	96.26
RF (LTC)	51.23	47.84	5.99	8.34	94.93
SVM (BTC)	49.07	52.16	7.86	19.69	127.13
SVM (ETH)	53.40	53.40	8.26	15.65	59.44
SVM (LTC)	54.32	50.93	11.96	33.28	144.60
*Test sample*					
Linear (BTC)	46.15	51.39	2.24	3.36	68.83
Linear (ETH)	53.85	54.46	3.65	5.20	80.60
Linear (LTC)	50.77	46.77	3.75	5.05	77.52
RF (BTC)	48.92	50.15	2.42	3.46	107.86
RF (ETH)	60.00	49.85	3.79	5.19	96.21
RF (LTC)	50.15	46.46	3.72	4.98	103.70
SVM (BTC)	51.08	50.15	2.98	4.25	625.61
SVM (ETH)	56.92	53.54	3.71	5.28	65.86
SVM (LTC)	55.69	59.69	3.59	4.98	43.87

This table shows some metrics aiming to assess the forecasting performance of all the proposed models: Linear models, Random Forests (RF) and Support Vector Machines (SVM). The Success Rate is the relative number of times that the model gives the right signal on the 1-day ahead return. This indicator is presented not only for the regression models, but also for their binary versions (Classification models). The other columns refer to the Mean Absolute Error (MAE), the Root Mean Square Error (RMSE) and the Theil’s U^2^. This last metric represents the ratio of the Mean Squared Error (MSE) of the proposed model to the MSE of a naïve model which predicts that the next return is equal to the last known return. All values are multiplied by 100

In the validation sub-sample, the success rates of the classification models range from 45.68% for the linear model applied to ethereum to 57.10% for the RF applied to bitcoin. Meanwhile, the success rates for the regression models range from 45.37% for the linear model applied to litecoin to 57.72% for the linear model applied to bitcoin. The success rate is lower than 50% in seven cases, with the linear classification model being the worst model class. During the validation period, the classification models produce, on average for the three cryptocurrencies, a success rate of 51.10%, which is slightly lower than the corresponding figure for the regression models (51.99%). In the validation sample, the MAEs range from 4.25 to 11.96%, and the RMSEs range from 6.85 to 33.28%. Two models, the SVM models for bitcoin and litecoin, are not superior to the naïve model, achieving a Theil’s U^2^ of 127.13% and 144.60%, respectively.

In the test sub-sample, the success rates of the classification models range from 46.15% for the linear model applied to bitcoin to 60.00% for the RF model applied to ethereum. Meanwhile, the success rates for the regression models range from 46.46% for the linear model applied to litecoin to 59.69% for the SVM model applied to litecoin. The success rate is lower than 50% in five cases, with the RF regression model being the worst model class. During the test period, the classification models produce, on average for the three cryptocurrencies, a success rate of 52.61%, which is slightly higher than the corresponding figure for the regression models (51.38%). In the test sample, the MAEs range from 2.24 to 3.79%, and the RMSEs range from 3.36 to 5.28%. The RF for bitcoin and litecoin and the SVM for bitcoin are not superior to the naïve model, achieving a Theil’s U^2^ of 107.9%, 103.7%, and 625.6%, respectively.

Given the unimpressive results of the models’ forecasting ability in the validation sub-sample and the positive empirical evidence on using model assembling (see e.g., Catania et al. 2019; Ji et al. 2019b; Mallqui and Fernandes 2019; Borges and Neves 2020), we analyze the performance of the trading strategies based on not the individual models but instead, their ensembles. Assembling the individual models also has an additional positive impact on the profitability of the trading strategies after trading costs, because it prescribes no trading when there is no strong trading signal; hence, reducing the number of trades and providing savings in trading costs. Table 7 presents the statistics on the performance of these trading strategies based on model assembling.

**Table 7:** Performance of the trading strategies on the test sample, based on model assembling

	B&H	Ensemble 4	Ensemble 5	Ensemble 6
*Bitcoin*				
Nº of days in the market (relative frequency in %)	325 (100%)	142 (43.69)	73 (22.46)	17 (5.231)
Win rate (%)	51.69	52.82	54.79	52.94
Average profit per day in the market (%)	− 0.2210	− 0.1892	0.0705	0.5356
SD of profit per day in the market (%)	3.271	3.600	3.814	4.351
Annual return (%)	− 54.86	− 25.74	5.868	10.61
Annual return with trading costs of 0.5% (%)	–	− 52.791	− 23.66	1.247
Annualized sharpe ratio (%)	− 129,1	− 66.44	16.83	54.95
Bootstrap *p*-value against B&H	–	0.0551	0.0269	0.0426
Daily CVaR at 1% (%)	11.60	9.443	8.070	3.882
Maximum drawdown (%)	67.17	48.06	30.94	11.15
*Ethereum*				
Nº of days in the market (relative frequency in %)	325 (100%)	113 (34.77)	56 (17.23)	30 (9.231)
Win rate (%)	46.15	53.98	60.71	63.33
Average profit per day in the market (%)	− 0.4048	0.0515	0.5951	0.8862
SD of profit per day in the market (%)	5.142	5.329	5.906	5.428
Annual return (%)	− 76.72	6.653	44.65	34.25
Annual return with trading costs of 0.5% (%)	–	− 28.35	9.622	14.35
Annualized sharpe ratio (%)	− 150.4	10.91	80.17	95.05
Bootstrap *p*-value against B&H	–	0.0140	0.0130	0.0278
CVaR at 1% (%)	17.81	13.40	12.63	7.661
Maximum drawdown (%)	89.67	45.86	28.92	14.40
*Litecoin*				
Nº of days in the market (relative frequency in %)	325 (100%)	103 (31.69)	53 (16.31)	12 (3.692)
Win rate (%)	46.46	51.46	50.94	50.00
Average profit per day in the market (%)	− 0.3025	0.1673	0.5094	0.0729
SD of profit per day in the market (%)	4.872	4.688	4.311	4.636
Annual return (%)	− 66.35	21.03	34.86	0.9746
Annual return with trading costs of 0.5% (%)	–	− 17.66	5.730	− 4.984
Annualized sharpe ratio (%)	− 118,7	38.48	91.35	6.025
Bootstrap *p*-value against B&H	–	0.0442	0.0546	0.1157
CVaR at 1% (%)	14.45	10.70	6.921	4.699
Maximum drawdown (%)	86.80	43.07	23.46	13.75

This table displays several statistics on the performance of the trading strategies in the test sample based on model assembling. The models considered are Linear, Random Forest (RF) and Support Vector Machine (SVM) and their binary versions, in a total of six models. Only long positions are considered, hence Ensemble 4, Ensemble 5 and Ensemble 6, refer to trading strategies designed upon the activation and maintenance of a long position when at least 4, 5 and 6 models agree on a positive trading sign for the next day, respectively. For clarity purposes, the table also presents, in the second column, the relevant statistics for the Buy-and-Hold (B&H) strategy. The trading signal for each model is obtained from the 1-step forecast using a rolling window with a constant length of 648 days. The win rate is equal to the ratio between the number of days when the ensemble model gives the right positive sign for the next day and the total of days in the market (previous line). The next two lines refer to the mean and standard deviation of the returns when the positions are active. The annual return is the compound return per year given by the accumulated discrete daily returns considering all days in the test sample, including zero-return days when the strategies prescribe not entering into the market. The next line refers to the compound return per year considering a proportional round-trip transaction cost of 0.5%. The Annualized Sharpe Ratio is the ratio between the daily mean return and the standard-deviation of daily returns considering all days in the test sample, multiplied by $\sqrt {365}$. The bootstrap *p*-values are the probabilities of the daily mean return of the proposed model, considering all days in the sample, being higher than the daily mean return of the Buy-and-Hold strategy that consists of being long all the time given the null that these mean returns are equal. These *p*-values are obtained using 100,000 bootstrap samples created with the circular block procedure of Politis and Romano (1994), with an optimal block size chosen according to Politis and White (2004) and Politis and White (2009). The CVaR at 1% measures the average loss conditional upon the fact that the VaR at the 1% level has been exceeded. Finally, the Maximum Drawdown is computed as the maximum observed loss from a peak to a trough of the accumulated value of the trading strategy, before a new peak is attained, relative to the value of that peak. All values are in percentage, except the nº of days in the market and the *p*-values.

The number of days in the market decreases roughly by half for Ensembles 4 and 5, and for Ensemble 6, the number of days in the market is marginal, never higher than 10%. The win rates are never lower than 50%, with the best results achieved by Ensembles 5 and 6 for ethereum, at 60.71% and 63.33%, respectively. The average profit per day in the market is negative only for Ensemble 4 for bitcoin; but in some other cases, it is quite low, not reaching 0.1%. However, all the strategies seem to beat the market (the daily mean returns of the B&H strategy are 0.22%, − 0.40%, and − 0.30%, for bitcoin, ethereum, and litecoin, respectively), except for Ensemble 6 for litecoin, where the bootstrap p-value of the mean daily return of the proposed strategy is higher than the mean daily return of the B&H strategy at around 0.12.

The annual returns are higher for Ensemble 5, as applied to ethereum and litecoin, achieving the values of 44.65% and 34.86%, respectively. These two strategies have impressive annualized Sharpe ratios of 80.17% and 91.35%. Ethereum stands out as the most profitable cryptocurrency, according to the annual returns of Ensembles 5 and 6, with and without consideration of trading costs. A possible explanation for this result is that ethereum is the most predictable cryptocurrency in the set, especially if those predictions are based not only on information concerning ethereum but also on information concerning other cryptocurrencies. Most studies that include in their sample the three cryptocurrencies examined here suggest that bitcoin is the leading market in terms of information transmission; however, some studies emphasize the efficiency of litecoin. For instance, Ji et al. (2019a) show that litecoin and bitcoin are at the center of returns and volatility connectedness, and that while bitcoin is the most influential cryptocurrency in terms of volatility spillovers, ethereum is a recipient of spillovers; thus, it is dominated by both larger and smaller cryptocurrencies. Bação et al. (2018) show that lagged information transmission occurs mainly from litecoin to other cryptocurrencies, especially in their last subsample (August 2017–March 2018), and Tran and Leirvik (2020) conclude that, on average, in the period 2017–2019, litecoin was the most efficient cryptocurrency.

All the strategies present a relevant tail risk, with the daily CVaR at 1% never dropping below 3%, and in some strategies achieving more than 10%. The maximum drawdown is never lower than 10%, even for Ensembles 6, where most of the days are zero-return days, whereas for Ensemble 4 (for the three cryptocurrencies), it is higher than 40%.

Naturally, the performance of the strategies worsens when trading costs are considered. With a proportional round-trip trading cost of 0.5%, the number of strategies that result in a negative annualized return increases from 1 to 5. However, most notably, the consideration of these trading costs highlights what is already visible from the other statistics, namely, that the best strategies are Ensemble 5 applied to ethereum and litecoin.

## Conclusions

This study examines the predictability of three major cryptocurrencies: bitcoin, ethereum, and litecoin, and the profitability of trading strategies devised upon ML, namely linear models, RF, and SVMs. The classification and regression methods use attributes from trading and network activity for the period from August 15, 2015 to March 03, 2019, with the test sample beginning at April 13, 2018.

For each model class, the set of variables that leads to the best performance is chosen according to the average return per trade during the validation sample. These returns result from a trading strategy that uses the sign of the return forecast (in the case of regression models) or the binary prediction of an increase or decrease in the price (in the case of classification models), obtained in a rolling-window framework, to devise a position in the market for the next day.

Although there are already some ML applications to the market of cryptocurrencies, this work has some aspects that researchers and market practitioners might find informative. Specifically, it covers a more recent timespan featuring the market turmoil since mid-2017 and the bear market situation afterward; it uses not only trading variables but also network variables as important inputs to the information set; and it provides a thorough statistical and economic analysis of the scrutinized trading strategies in the cryptocurrencies market. Most notably, it should be emphasized that the prices in the validation period experience an explosive behavior, followed by a sudden and meaningful drop; nevertheless, the mean return is still positive. Meanwhile in the test sample, the prices are more stable, but the mean return is negative. Hence, analyzing the performance of trading strategies within this harsh framework may be viewed as a robustness test on their profitability.

The forecasting accuracy is quite different across models and cryptocurrencies, and there is no discernible pattern that allows us to conclude on which model is superior or which is the most predictable cryptocurrency in the validation or test periods. However, generally, the forecasting accuracy of the individual models seems low when compared with other similar studies. This is not surprising because the best in-class model is not built on the minimization of the forecasting error but on the maximization of the average of the one-step-ahead returns. The main visible pattern is that the forecasting accuracy in the validation sub-sample is lower than in test sub-sample, which is most probably related to the significant differences in the price trends experienced in the former period.

Taking into account the relatively low forecasting performance of the individual models in the validation sample, and the results already reported in the literature that model assembling gives the best outcomes, the analysis of profitability in the cryptocurrencies market is conducted considering trading strategies in accordance with the rules that a long position in the market is created if at least four, five, or six individual models agree on the positive trading sign for the next day. The trading strategies only consider the creation of long positions, given that short selling in the market of cryptocurrencies may be difficult or even impossible. This restriction is quite binding, as the test period is characterized by bearish markets, with daily mean returns lower than − 0.20%.

The win rates of the strategies are never lower than 50%, with the best results achieved by Ensembles 5 and 6 for ethereum, at 60.71% and 63.33%, respectively, but the mean daily returns are not impressively high. Generally, these strategies are able to significantly beat the market. Additionally, these trading strategies are subjected to a high tail risk, with CVaRs at 1% between 3.88% and 13.40% and maximum drawdown between 11.15% and 48.06%. Basically, the results point out that the best trading strategies are Ensemble 5 applied to ethereum and litecoin, which achieved an annualized Sharpe ratio of 80.17% and 91.35% and an annualized return, after proportional trading costs of 0.5%, of 9.62% and 5.73%, respectively. These values seem low when compared with the daily minima and maxima returns of these cryptocurrencies during the test sub-sample. However, one may argue that the fact that they are positive may support the belief that ML techniques have potential in the cryptocurrencies market, that is, when prices are falling down, and the probability of extreme negative events is high, the trading strategy still presents a positive return after trading costs, which may indicate that these strategies may hold even in quite adverse market conditions.

It is noteworthy that in ML applications there are many decisions to be made concerning the best methods, data partitioning, parameter setting, attribute space, and so on. In this study, the main goal is not to test extensively the alternative forecasting and trading strategies; hence, there is no guarantee that we are using the best methods available. Instead, our aim is more modest, as we simply try to figure out if ML can, in general, lead to profitable strategies in the cryptocurrency market and if this profitability still exists when market conditions are changing and more realistic market features are considered. Higher frequency data, for instance using real transaction prices from a particular online exchange; a wider input set including more refined attributes such as technical analysis indicators; the consideration of bitcoin futures, where short positions are easily created and transaction costs are lower—all these arguably may lead to better results.

## Data Availability

The data used in this research paper are public, obtained from the CoinMarketCap site (https://coinmarketcap.com/) and from the Coin Metrics site (https://coinmetrics.io/).
